# Bacterial aerosol, particulate matter, and microclimatic parameters in the horse stables in Poland

**DOI:** 10.1007/s11356-021-18142-6

**Published:** 2021-12-18

**Authors:** Jacek Grzyb, Zenon Podstawski, Karol Bulski

**Affiliations:** 1 Department of Microbiology and Biomonitoring, University of Agriculture in Kraków, Mickiewicza Ave 24/28, 30-059 Kraków, Poland; 2 Department of Reproduction, Anatomy and Genomics of Animals, University of Agriculture in Kraków, Mickiewicza Ave 24/28, 30-059 Kraków, Poland

**Keywords:** Stable, Airborne bacteria, Particulate matter, Size distribution, Bioaerosol exposure

## Abstract

Keeping horses in good condition requires providing them with living conditions that meet welfare requirements. These animals should be accommodated with suitable space, access to high nutritional fodder and water, and a suitable microclimate in their shelters. When it comes to the environment in the stables, a serious problem is created by particulate matter (PM), which consists of bacteria. PM concentration may be responsible for developing multiple lower respiratory tract diseases in horses, including allergies and recurrent airway obstruction (RAO). In turn, these ailments may lead to decreasing equine physical and mental fitness. Additionally, people who spend time in the stables are exposed to the same harmful factors. The study was conducted in Udórz Stud Farm located in the southern region of Poland. The study was carried out in 2 different types of stables: 3 runners (a type of stable where horses are housed together and occasionally linked up, e.g., for feeding or grooming) and 2 box stables. The research continued for 2 years and the samples were collected in each season. The bioaerosol samples were collected using a six-stage Andersen-Graseby cascade impactor to assess size distribution and concentrations of airborne bacteria. PM concentration was analyzed using the DustTrak™ II Aerosol Monitor 8530, while microclimate parameters were measured using the Kestrel 5000 Weather Meter. There are almost no studies concerning size distribution of airborne bacteria, individual PM fractions, and the impact of everyday handling on the changes in the bioaerosol and PM concentration. This preliminary study provided basic information on this subject. We have revealed a strong correlation between high PM and bacterial aerosol concentrations. Higher contamination levels were recorded in runners, as compared to box stables. The highest bacterial aerosol level was detected in the spring. The analysis of the fractions of the bacterial aerosol in the stables indicated the highest share of ultra-fine fraction (0.65–2.1 µm), while respirable fraction (below 4.7 µm) exceeded 75%. It was established that the concentration of the bacterial aerosol inside the stables was many times higher than outside. It depended significantly on everyday activities undertaken in the stables, like feeding or cleaning. Taking the above into account, a different cleaning system should be developed (a wet cleaning system, with the use of water) and excrement should be removed more frequently.

## Introduction

Living conditions created for horses should meet welfare requirements, which ensures keeping horses in good health. It requires implementing guidelines on animal hygiene and environmental sanitation, in particular with regard to maintenance, feeding, and equipping stables. The requirements established for horses were defined in the Regulation of the Minister of Agriculture and Rural Development (J.L. of 2018, item 116). This document provides detailed guidelines on horse maintenance systems, including requirements that must be met with regard to stables. Two types of equine management systems (among 4 available, that is, box stables, runners, stationary stables, and free-range stables) are used in the research facility: box stables and runners. A box system is usually adopted for mares, stallions, sports horses, and riding horses. The size of the box should enable free movement and the possibility of lying down. As the horses are herd animals, it is more natural to apply a free stall housing system—in stables called runners. Runners are used for young horses, infertile mares, and mares with foals. According to the abovementioned Regulation, horses in the stables should be provided with bedding. Bedding inside the stable should absorb moisture and protect the horse’s leg from contact with a hard surface. Good-quality bedding enables reaching a suitable hygiene level, affects stables microclimate, and facilitates keeping horses clean (Kołacz and Dobrzański 2019; Waran 2007; Łojek et al. 2009).

A stable is also a place where many people spend quite a lot of time—either as employees involved in taking care and training horses or enthusiasts devoting their free time. We must be aware that all people staying in the stables, as well as horses themselves, are exposed to inhaling particulate matter. Particulate matter (PM) consists of organic components, such as saprophytes and pathogenic bacteria, spores, mites remains, plant remains, and inorganic dust. PM concentration in the air inside the stable depends on the type of bedding, fodder provided to animals, animal-associated microbes, and their feces (Siegers et al. 2018). PM concentration is closely related to the bioaerosol concentration, as PM represents a major factor responsible for transferring biological particles in the air (Wolny-Koładka 2018).

PM components found in the stable air may cause respiratory tract inflammation in horses through triggering allergy, infection, or indirectly via overloading the pulmonary defense mechanism and a disease called recurrent airway obstruction (RAO) (Witkowska et al. 2012).

As far as the aerodynamic diameter of PM particles is concerned, PM can be divided into 2 fractions: particles with the diameter exceeding 4.7 µm that are capable of depositing in the upper respiratory tract (inhalable) and respirable, including particles with the diameter lower than 4.7 µm. In the case of humans, particles classified as respirable fraction penetrate into the lower respiratory tract, what causes inflammation and irritation. When it comes to horses, the exact size of bioaerosol particles that are capable of migrating into particular respiratory tract compartments is not known. It is assumed that respirable fractions play a major role in the pathogenesis of asthma and RAO in horses (Fleming et al. 2008; Ivester et al. 2014; Pirie et al. 2016). Additionally, it is believed that respirable fractions of both PM and bioaerosol, that is, with the aerodynamic diameter below 5 µm, reach the same locations in the airways of both horses and other animals (Clements and Pirie 2007; Hessel et al. 2009; Auger and Moore-Colyer 2017).

Employees responsible for handling horses may also experience health problems associated with inhaling PM. An increased incidence of asthma and decreased respiratory capacity was observed in the case of grooms (Wälinder et al. 2011).

Admissible microorganism concentrations in the air inside livestock facilities have not been established yet—only the recommendations were issued (Gołofit-Szymczak and Górny 2010). Many factors contribute to generating high PM concentrations inside the stables, and thus bacterial aerosol. Major ones include the following: age and type of the building, type of ventilation, facility size, livestock density, type of bedding, type of feed, and microclimate conditions (Witkowska et al. 2012).

The study was aimed at establishing how different horse management systems affect the concentration of PM, bacteria, and microclimate conditions inside the stables. It will allow for determining potential health risks for horses, grooms, and equine enthusiasts.

## Materials and methods

**Fig. 1:**
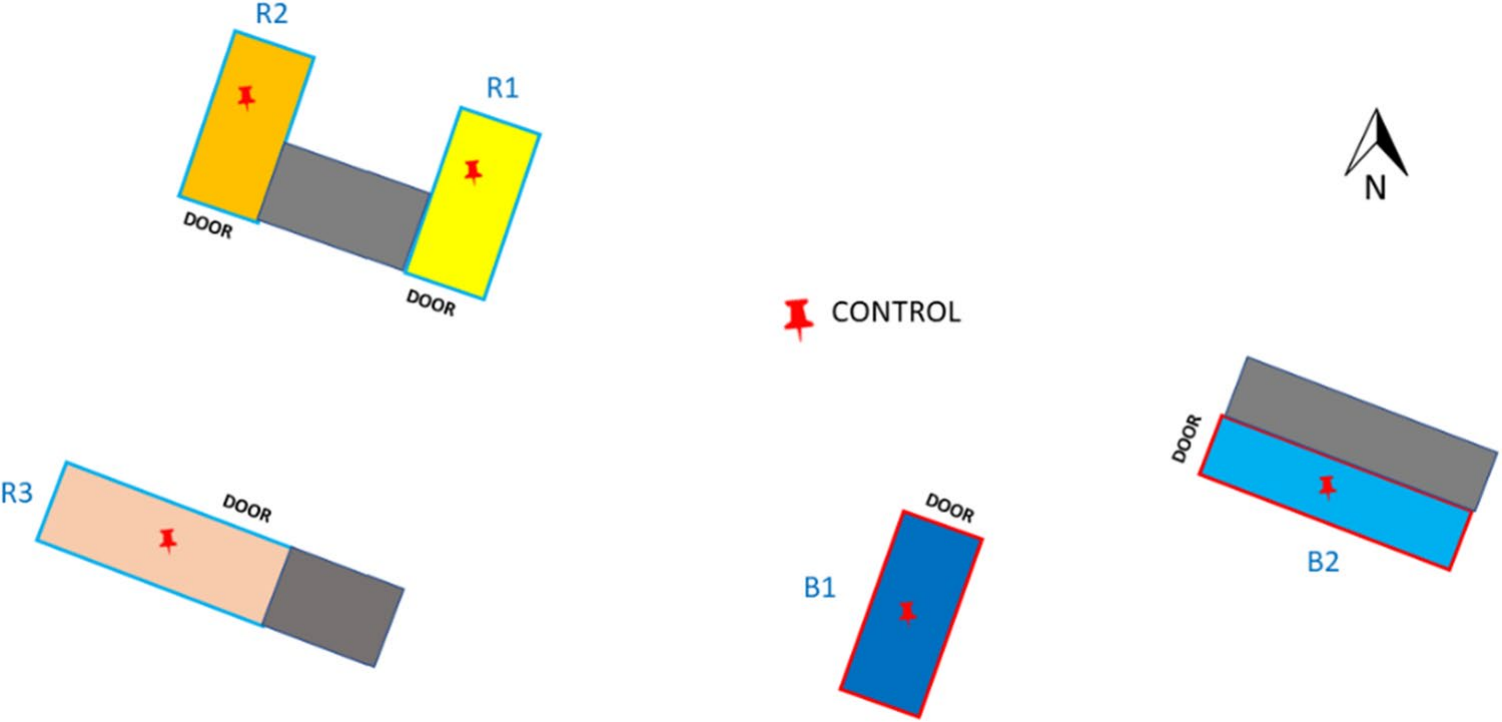
Scheme of the arrangement of the stables with sampling sites. Legend: R1–R3 runners, B1, B2 box stables, CONTROL control site, red pushpin sampling point

The study was conducted in Udórz Stud Farm. The horse stud is located in the southern region of Poland, in the Silesian Province, in Zawiercie County. Four stables are located within the stud farm area. The first building includes 2 runners (R1 and R2), the second one runner (R3), and next two buildings include box stables (B1 and B2) (Fig. 1). The control site (C) was located outside the stables, within the stud farm area in the distance of at least 50 m from the closest stable. During conducting the study, 84 horses were maintained on the stud farm. Particular horse groups and their age are presented in Table 1.

**Table 1:** Characteristics of the studied premises

Parameter	Type of stable				
	Runner 1 (R1)	Runner 2 (R2)	Runner 3 (R3)	Box stable 1 (B1)	Box stable 2 (B2)
Year of construction	1955	1955	1935	1976	1995
Type of ventilation	Gravity ventilation				
Type of litter	Straw				
Cleaning frequency	All manure is removed once a month; a new layer of straw every day			All manure is removed once a week; daily cleaning up of feces	
Cleaning technique	Removing manure with a front loader				
Group of horses	12 mares 5 foals	4 mares, 3 young mares (2 years old) 5 young mares (1 year old)	5 stallions 7 young stallions (2 years old) 6 young stallions (1 year old)	Sport horses: 7 mares 10 geldings	Sport horses: 9 mares 11 geldings
Number of horses	17	12	18	17	20
Total area (m^2^)	283.8	281.6	386.8	395.5	450.3
Height (m)	3.95	3.95	4.0	3.5	3.6
Volume (m^3^)	1121.01	1112.32	1545.6	1384.25	1621.08
Surface to volume ratio	71.8	71.3	96.7	113.0	125.1
Area per 1 animal (m^2^)	16.69	23.47	21.49	23.26	22.52
Volume per 1 animal (m^3^)	65.92	92.7	85.96	81.4	81.07
Mean animal weight (kg)	417.6	387.5	441.66	583.00	575.00
Total animal weight (kg)	7099.2	4650.0	7949.8	9911.0	11,500.0
Livestock units (LU)*	14.2	9.3	15.9	19.8	23.0
Ratio – kg of animal weight per 1 m^2^ of area	25	16.5	20.6	25	25.5
Ratio – kg of animal weight per 1 m^3^ of volume	6.3	4.2	5.1	7.2	7.1

Legend: *Livestock unit (LU) = standardized to an animal weight of 500 kg

During the wintertime, windows in the stables were open. Windows were left open overnight or closed if there was a significant temperature drop. From spring (after the frosts have subsided) to autumn (until the first frosts), windows were open. During the winter season, the upper half of the door was open, while from spring to autumn, the door was fully open. The horses were kept indoors only at night. During the day, the circular (pasture) was used: in the winter from 7.00 AM to 04.00 PM, in the spring from 7.00 AM to 06.00 PM, in the summer from 6.00 AM to 09.00 PM, and in the autumn from 06.00 AM to 06.00 PM. Sport horses spent the most time in stables, making exercise only in lunging or in circulars (about 2 h a day) and during training.

The horses were fed with fodder obtained from the farm’s crops. Older horses and foals were fed with oats (whole and crushed grain), hay, and (during the vegetation season) grass from pastures. After the grazing season, horses were supplemented with a macro- and microelement mixture with vitamins. Lactating mares and foals were supplemented with wheat bran. The horses had permanent access to water and salt licks. Sport horses were fed with oats with the addition of industrial mixtures dedicated to sport horses—the doses of mixtures were calculated on the basis of horse nutrition standards [Brzóska et al. 2015]. The horses were fed differently, mostly due to the way of their use. Stallions, sport, and recreational horses (kept in box stables) received doses calculated strictly according to the needs. Lactating mares and foals (kept in the runners) received a standard dose of oats and bran, as well as permanent access to hay in the winter. In the summer, they used the pasture. Foals in runners had permanent access to concentrated fodder (crushed oats and bran) and hay. There was a difference in feeding between horses in box stables and runners—the feeding times during the day were different. The horses in box stables were fed with concentrated fodder three times a day (in the morning, noon, and evening). Breeding horses were fed with concentrated fodder twice a day (in the morning and evening), and in the meantime they used pasture.

Straw bedding was used in the studied facilities. A fresh layer of straw was added every day in both stable types. Old straw bedding was replaced at different frequency: on average, once a month in runners, and once per week in box stables.

The measurements were taken throughout the whole 2 calendar years—once each season (spring – April, summer – July, autumn – September, winter – February). Due to the inability to perform the tests in a continuous manner in each month, it was found that during these months, there is the most typical weather for a given season.

The research facilities were selected based on the following criteria:

- type of the stable (box stable, runner),
- impact of the season.

Air samples were taken using a 6-stage cascade impactor WES-710 model Andersen-Graseby (Westech Instrument, Great Britain). This impactor enables to determine bioaerosol fractions based on the aerodynamic particle size: F1, above 7 µm; F2, 4.7–7 µm; F3, 3.3–4.7 µm; F4, 2.1–3.3 µm; F5, 1.1–2.1 µm; and F6, 0.65–1.1 µm. Fractions 3–6 (below 4.7 µm) are classified as respirable (called RF)**.**

The samples were taken about the same time. The first measurement series were taken between 7.00 and 10.00 AM—before feeding and stable cleaning. The second approximately 1 h after cleaning the stables, feeding horses, and adding fresh bedding (11.00 AM–2.00 PM).

The sampling sites were located in the middle of each stable. Air samples were collected 1.5 m above the ground, to collect the air from the human and horses breathing zone. Six Petri dishes were used to collect the samples—one for each impactor stage. The time necessary to collect the samples depended on anticipated concentrations of bacteria in a given location. The flow rate through the impactor was constant and amounted to 28.3 dm^3^/min. The samples were collected within 10 to 30 s, and the volume of aspirated air ranged between 4.7 and 14.1 dm^3^. The measurement in the control site—outside the buildings—took from 120 to 240 s (56.6–113.2 dm^3^), depending on the season. The impactor was disinfected using gauze pads moistened in 70% isopropanol before taking each sample.

TSA medium (Tripticasein Soy Lab Agar, Biomaxima Polska), for the culturing of bacteria, was used in the study. Media were incubated under the following conditions: 1 day at 37 °C, then 3 days at 22 °C and 3 days at 4 °C under aerobic conditions. Prolonged incubation was aimed at enabling the growth of slow strains at a lower temperature range (Jensen and Schafer 1998). Incubation parameters were selected to enable growth of bacteria in a wide optimal temperature range. After the incubation, the colonies were counted and the results were expressed as colony forming units per 1m^3^ of air (CFU/m^3^). The concentrations of bacteria were calculated according to the following formula: L = [Pr · 1000]/v, where L – concentration of microorganisms in 1 m^3^ of air, Pr – probable statistical count according to the impactor manufacturer’s table (Pr is read from the table on the basis of the number of colonies), v – volume of air taken by the impactor (dm^3^), 1000 – converter to 1 m^3^. The tests were performed in triplicate and the results were presented as the means.

The recorded bioaerosol concentrations, due to the absence of guidelines on the acceptable concentrations of microorganisms in stables, were referred to the proposal of the Team of Experts in Biological Factors (Polish; ZECB) (Augustyńska and Pośniak 2016) on the recommended concentrations of airborne microorganisms, treating stables as working premises contaminated with organic dust (Table 2).

**Table 2:** Proposals for acceptable concentrations of bacteria according to the Team of Experts in Biological Factors (ZECB)

Microbiological agent	Acceptable concentration (CFU/m^3^)
Total count of bacteria (TC)	100,000
Respirable fraction of bacteria (RF)	50,000

PM concentrations were measured using a DustTrak™ II Aerosol Monitor 8530 (TSI Inc., USA) laser photometer. The device allows to measure 4 fractions of PM: PM_10_ (i.e., PM particles not larger than 10 μm), PM_4_, PM_2.5_, and PM_1_ (PM particles with diameters below 4, 2.5, and 1 μm, respectively) using interchangeable heads. The sampling time for each PM fraction was 180 s, with a sampling time of every 3 s, which gave a total of 60 independent measurements. Microclimatic parameters (temperature and relative humidity) were measured using the Kestrel 4000 Weather Meter (Nielsen-Kellerman, USA).

Statistical analysis of the data was performed using software Statistica, version 13.1 – 2018 (StatSoft, Inc., Tulsa, OK, USA). After taking into account the fulfillment of the assumptions about the normality of the distribution of variables (Shapiro–Wilk’s test) and the homogeneity of variance (Levene’s test), the analysis of variance was performed (ANOVA and two-way ANOVA), and the significance of differences between the means was verified with the Tukey’s test. The values for which the probability “p” was lower than 0.05 were considered as statistically significant. The impact of microclimatic parameters (air temperature and relative humidity) and PM on the quantitative presence of bacteria in the air was assessed using Pearson’s correlation coefficient for the studied dependencies, assuming statistically significant values at *p* < 0.05 (Bulski et al. 2019; Wolny-Koładka et al. 2017).

## Results

Due to the absence of standards, the results obtained in the study were analyzed against recommendations issued by ZECB presented in Table 2. The stables are treated as “working premises contaminated with organic dust.” On that basis, permissible concentrations were exceeded in the case of 20% of measurements for the total concentration of the bacterial aerosol (TC), while—for the respirable fraction (RF)—it concerned 28.7% of the measurements taken (Table 3). An allowable bacteria concentration was exceeded most frequently in the spring (35% of the measurements for TC and 40% for RF), three times more frequently within runners as compared to box stables. Significantly more measurements were taken after cleaning the stables (“after bedding”; AB), when permissible concentrations were exceeded, as compared with the measurements made before replacing bedding (“before bedding”; BB): TC 37.5 vs 2.5%, while for RF 42.5 vs 15%.

**Table 3:** The degree of exceeding the permissible concentrations of bacteria in relation to the ZECB guidelines (%)

Factor		Fraction	
		TC	RF
Total		20	28.7
Season	Spring	35	40
	Summer	25	30
	Autumn	10	20
	Winter	10	25
Bedding	AB	2.5	15
	BB	37.5	42.5
Stable	Runner	33.3	39.6
	Box stable	9.4	12.5

Legend: *TC* total count of bacteria, *RF* respirable fraction, *BB* before bedding, *AB* after bedding

As shown in Table 4, the highest average concentration of the bacterial aerosol BB, within runners, was detected in stable R1 (43,782 CFU/m^3^), while AB in R2 (181,132 CFU/m^3^). The lowest bioaerosol concentration was recorded in box stables: BB in stable B2 (23,997 CFU/m^3^), AB in stable B1 (45,911 CFU/m^3^). The assessment of the mean bioaerosol concentration according to the type of stable revealed that higher bacterial contamination occurred in runners (R1–R3), as compared with box stables, both BB (1.5 times) and AB (2.25 times). After cleaning the stable, the concentration of bacterial aerosol increased 3.5 times for runners, and 2.4 times for box stables (B1 and B2). As compared against the control site (C), the bacterial concentration within runners BB was 24 times higher on average, while for box stables 16 times higher, and AB, 85 vs 38 times higher, respectively. The statistical analysis of the TC of the bacterial aerosol (without taking into account the season, tab. 4) showed significant differences between BB and AB only in the R2 measuring point (Tukey’s test, *p* < 0.05). There were also statistically significant differences in the concentration of bacteria between the control sample (C) and R1 AB and R2 AB measuring points (Tukey’s test, *p* < 0.05).

**Table 4:** Average, standard deviation, range, and total concentrations (TC) of bacteria in stables (CFU/m^3^)—before (BB) and after bedding (AB) (CFU/m^3^)

Stable	Bedding	Average ± standard deviation	Range	Total	*p-value*
R1	BB	43,782ab** ± 47,418	12,425–152,853	350,259	> 0.05
	AB	133,763bc ± 130,460	11,453–348,350	1,070,106	
R2	BB	27,170ab ± 18,056	4134–53,449	217,362	0.029
	AB	181,132c ± 171,797	22,578–541,872	1,449,056	
R3	BB	41,680ab ± 22,415	16,117–74,783	333,441	> 0.05
	AB	81,535abc ± 51,804	1908–166,670	652,280	
**All R**	BB	**37,544a ± 31,518**	**4134–152,853**	**901,063**	0.039
	AB	**132,143bc ± 129,251**	**1908–541,872**	**3,171,442**	
B1	BB	24,730ab ± 25,308	8201–85,406	197,838	> 0.05
	AB	45,911ab ± 30,920	22,957–121,030	367,287	
B2	BB	23,997ab ± 14,447	3221–44,753	191,977	> 0.05
	AB	71,512abc ± 50,713	26,251–143,032	572,097	
**All B**	BB	**24,363ab ± 19,911**	**3221–85,406**	**389,815**	> 0.05
	AB	**58,711ab ± 42,674**	**22,957–143,032**	**939,384**	
C		1554a ± 1103	612–4040	12,432	

Legend: *BB* before bedding, *AB* after bedding, *C* control; *R1*, *R2*, *R3* runner 1, runner 2, runner 3; *B1* box stable 1, *B2* box stable 2, *All R* average for all runners, *All B* average for box stables, *C* control

^**^Averages marked with the same letters are not significantly different by Tukey’s test (*α* = 0.05)

Table 5 shows the concentrations of bacterial aerosol taking into account the classification based on the aerodynamic diameter of the particles. Depending on the bioaerosol fraction and the type of stables, an increase reached the values from 232% for F6 fraction (0.65–1.1 µm) in runners, to as much as 522% for F3 fraction (4.7–3.3 µm) also in runners. The TC of bioaerosol increased more significantly (by 111%) in runners, as compared to box stables. The difference for the RF was even higher and amounted to as much as 128%. The highest increase in the bioaerosol concentration in box stables AB was recorded for fraction F2 (7.0–4.7 µm)—by 346%. A statistical analysis of the variability of the concentration of individual bacterial aerosol fractions (tab. 5) showed a statistically significant increase in the concentration of bacteria in each tested fraction (F1–F6) at points runners R1–R3 AB, compared to the concentration of the bacterial aerosol at these points BB (Tukey’s test, *p* < 0.05). At measuring points boxes B1–B2, there was an increase in the concentration of each tested bacterial aerosol fraction (F1–F6) AB compared to the bacteria concentration BB, but the increase was not statistically significant (Tukey’s test, *p* > 0.05). Analogous results of the statistical analysis were observed for TC and RF for points R1–R3 and B1–B2.

**Table 5:** Average, standard deviation, and range of bacteria concentration in stables—split by fraction

Stable	Fraction (µm)	Average ± standard deviation	Range (min–max)	Average ± standard deviation	Range (min–max)	Change of concentrations—BB vs AB*** (%)	*p-value*
Runner (R1 – R3)		BB		AB			
	F1	5334a**** ± 4278	867–21,624	13,970b ± 13,186	212–52,286	262%	0.041
	F2	3123a ± 2619	106–12,797	14,493b ± 14,847	106–55,668	464%	0.038
	F3	3930a ± 3454	212–12,160	20,516b ± 24,065	212–109,180	522%	0.031
	F4	5720a ± 5415	497–21,045	26,973b ± 29,948	1166–126,034	472%	0.030
	F5	8593a ± 11,071	636–53,379	31,022b ± 37,978	0–154,336	361%	0.033
	F6	10,845a ± 11,837	530–58,540	25,170b ± 26,181	212–99,654	232%	0.040
	TC	37,544a ± 31,518	4134–152,853	132,143b ± 129,251	1908–541,872	352%	0.039
	RF	29,088a ± 28,392	1908–134,189	103,681b ± 107,707	1590–457,602	356%	0.039
Box stables (B1—B2)		BB		AB			
	F1	2940a ± 1909	407–8374	7353a ± 6948	1838–22,048	250%	> 0.05
	F2	2199a ± 2054	495–8268	7613a ± 6757	1131–25,800	346%	> 0.05
	F3	2719a ± 2754	213–10,888	8824a ± 8691	2121–36,852	325%	> 0.05
	F4	4213a ± 4025	142–16,897	9801a ± 7781	1555–25,420	233%	> 0.05
	F5	5574a ± 6347	655–27,290	11,437a ± 8463	3651–28,408	205%	> 0.05
	F6	6719a ± 6276	655–25,876	13,683a ± 11,103	3690–45,850	204%	> 0.05
	TC	24,363a ± 19,911	3221–85,406	58,711a ± 42,674	22,957–143,032	241%	> 0.05
	RF	19,225a ± 18,093	2230–76,710	43,745a ± 31,181	17,158–106,530	228%	> 0.05
Control (C)	F1	278 ± 249	82–817				
	F2	173 ± 178	47–540				
	F3	170 ± 175	35–582				
	F4	197 ± 196	53–667				
	F5	341 ± 236	47–696				
	F6	395 ± 202	106–781				
	TC	1554 ± 1103	612–4040				
	RF	1103 ± 722	458–2684				

Legend: Fractions, F1 = 11.0–7.0 µm, F2 = 7.0–4.7 µm, F3 = 4.7–3.3 µm, F4 = 3.3–2.1 µm, F5 = 2.2–1.1 µm, F6 = 1.1–0.65 µm

*TC* total count of bacteria; *RF* respirable fraction

^***^Computed in relation to the average

^****^Averages marked with the same letters are not significantly different by Tukey’s test (α = 0.05), separately for each fraction between before (BB) and after bedding (AB)

The comparison of the mean concentration for individual bioaerosol fractions (Table 5) and percentage share of individual fractions (Table 6) revealed that, irrespective of the type of stables, the highest bacterial concentrations were detected for fine fractions, mainly fraction F6. Percentage share for that fraction BB runners and box stables amounted to 28.9% and 28.5%, and AB to 19.0% and 20.0%, respectively (Table 6). A decrease in the proportion of the finest fraction AB means that particles that float into the air include bacteria mainly from the range 2.1–4.7 µm (fractions F3–F4). The lowest bacteria concentrations and percentage shares were recorded BB for the fraction F2 and amounted to 8.3% and 8.5% in runners and box stables, while AB for fraction F1 10.6% and 11.0%, respectively.

**Table 6:** The share of the bacterial aerosol fraction (%) in stables: before (BB) and after bedding (AB)

Stable	Bedding	Bioaerosol fraction (RF = F3 ÷ F6)						
		F1	F2	F3	F4	F5	F6	RF
R1	BB	13.1	8.6	10.6	15.2	23.6	28.8	78.3
	AB	9.7	15.4	16.9	16.5	19.1	22.4	74.9
R2	BB	13.4	8.5	10.1	14.4	24.4	29.2	78.1
	AB	10.2	12.3	15.8	18.6	23.4	19.7	77.5
R3	BB	13.9	8.4	10.4	14.9	23.5	28.8	77.6
	AB	10.5	11.0	15.5	20.0	24.2	18.9	78.5
**All R**	BB	**14.2**	**8.3**	**10.5**	**15.2**	**22.9**	**28.9**	**77.5**
	AB	**10.6**	**11.0**	**15.5**	**20.4**	**23.5**	**19.0**	**785**
B1	BB	13.8	8.5	10.6	15.7	22.9	28.6	777
	AB	10.5	11.0	15.4	20.3	23.3	19.4	78.5
B2	BB	13.6	8.5	10.7	15.8	22.9	28.5	77.9
	AB	10.8	11.2	15.4	19.8	22.9	19.9	78.0
**All B**	BB	**13.6**	**8.5**	**10.7**	**15.9**	**22.9**	**28.5**	**77.9**
	AB	**11.0**	**11.4**	**15.4**	**19.6**	**22.6**	**20.0**	**77.6**
**C**		**13.5**	**8.6**	**11.2**	**16.0**	**22.5**	**28.2**	**77.9**

Legend: Fractions, F1 = 11.0–7.0 µm, F2 = 7.0–4.7 µm, F3 = 4.7–3.3 µm, F4 = 3.3–2.1 µm, F5 = 2.2–1.1 µm, F6 = 1.1–0.65 µm

*RF* respirable fraction; *BB* before bedding; *AB* after bedding; *R1*, *R2*, *R3* runner 1, runner 2, runner 3; *B1* box stable 1; *B2* box stable 2; *All R* average for all runners; *All B* average for box stables; *C* control

**Fig. 2:**
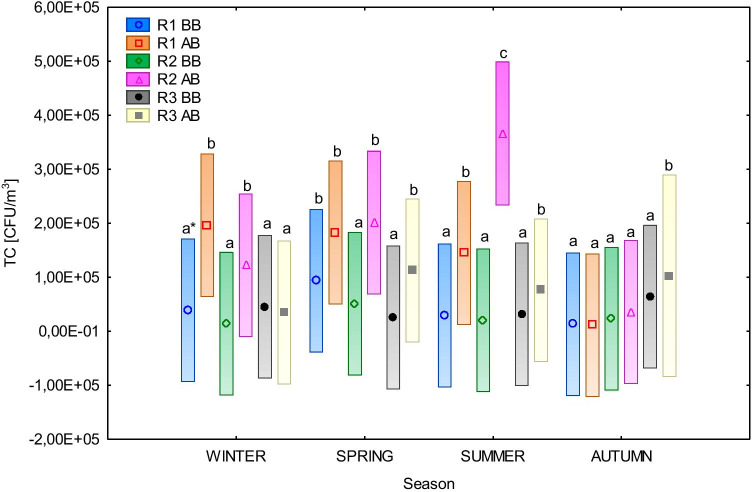
The significance of differences in average total bacterial aerosol concentration (TC) (CFU/m^3^) in runners (R1–R3) – before (BB) and after bedding (AB) between the seasons (two-way ANOVA, vertical bars represent confidence intervals .95). * averages marked with the same letters are not significantly different by Tukey’s test (*α* = 0.05)

**Fig. 3:**
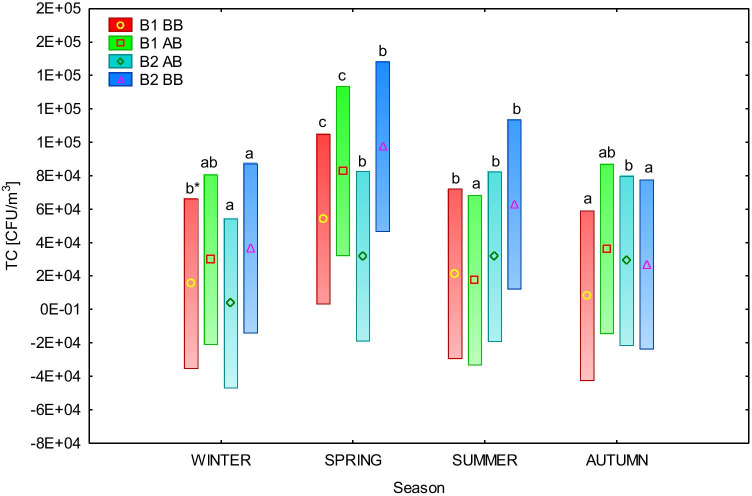
The significance of differences in average total bacterial aerosol concentration (TC) (CFU/m^3^) in box stables (B1–B2) – before (BB) and after bedding (AB) between the seasons (two-way ANOVA, vertical bars represent confidence intervals .95). * averages marked with the same letters are not significantly different by Tukey’s test (*α* = 0.05)

The statistical analysis of the TC of the bacterial aerosol showed significant differences in the concentration at measuring points between the seasons (Fig. 2, Fig. 3). In the case of runners (R1–R3), statistically significant differences in the concentration of bacteria between the seasons were noted at points R1 BB, R1 AB, R2 AB, and R3 AB (Tukey’s test, *p* < 0.05). At points R2 BB and R3 BB, no significant differences in the concentration of bacterial aerosol between the seasons were found (Tukey’s test, *p* > 0.05). The highest significant statistical differences in the concentration of bacteria between the seasons were noted at point R2 AB (summer–autumn). In the case of box stables, statistically significant differences in the concentration of bacteria between the seasons were noted at all measuring points (B1 BB, B1 AB, B2 AB, B2 BB) (Tukey’s test, *p* < 0.05). The highest significant statistical differences in the concentration of bacteria between the seasons were detected in point B2 BB (spring–autumn).

The analysis of the proportion of the RF in the TC of bacterial aerosol depending on the season (Table 7) indicated that the highest proportion of RF occurred in the autumn in all runners (82–86.8%) and B2 (84.1%). In the stable B1, the highest proportion of RF in TC was detected in the spring (88.0%). The lowest content proportion for RF was obtained for most research facilities in the summer (R1, R3, B1), and for the remaining ones (R2 and B2) in the winter.

**Table 7:** Bacterial aerosol: RF share in TC depending on the season (%)

Stable	Season			
	Spring	Summer	Autumn	Winter
R1	83.3	63.8	86.8	76.2
R2	80.8	81.9	83.4	72.8
R3	75.3	72.8	82.0	80.1
**All R**	**79.8**	**72.8**	**84.0**	**76.4**
B1	88.0	63.9	79.0	70.7
B2	73.1	70.1	84.1	72.4
**All B**	**80.6**	**67.0**	**81.5**	**71.5**
**C**	**72.7**	**59.2**	**88.0**	**66.1**

Legend: *TC* total count of bacteria, *RF* respirable fraction; *R1*, *R2*, *R3* runner 1, runner 2, runner 3; *B1* box stable 1; *B2* box stable 2; *All R* average for all runners; *All B* average for box stables; *C* control

The assessment of the air quality inside the stables covers the occurrence of bacterial intoxication. This phenomenon happens when the concentration of bacteria indoors (I) is higher than outdoors (O) (Table 8). Especially high intoxication level was observed in the stable R2 AB in the summer; the bacterial concentration inside this facility was 380-fold higher compared to the control site. The lowest intoxication level was recorded in the winter in the stable B2 AB (1.5 ×). The lowest average intoxication occurred in the stable B2, while the highest in the stable R2. The average intoxication for all measurements BB amounted to 22.8, while in the case of measurements taken AB, the intoxication level increased more than threefold and reached the value of 76.5. The average intoxication level for runners amounted to 62 and was almost twofold higher than in box stables.

**Table 8:** Inside/outside ratio (I/O) depending on the season and the horse care system

Stable	Season								Average
	Spring		Summer		Autumn		Winter		
	BB	AB	BB	AB	BB	AB	BB	AB	
R1	55.2	107.8	30.5	150.2	11.1	9.7	16.4	82.5	57.9
R2	30.1	118.5	21.2	379.5	19.5	30.2	5.9	51.4	82.0
R3	15.0	66.3	32.9	79.0	54.3	87.0	19.1	14.7	46.0
**All R**	**33.4**	**97.6**	**28.2**	**202.9**	**28.3**	**42.3**	**13.8**	**49.5**	**62.0**
B1	31.8	48.8	22.2	36.1	7.0	30.7	6.5	12.5	24.5
B2	18.8	57.3	32.8	130.2	24.6	22.8	1.5	15.3	37.9
**All B**	**25.3**	**53.1**	**27.5**	**83.2**	**15.8**	**26.7**	**4.0**	**13.9**	**31.2**

Legend: *BB* before bedding; *AB* after bedding; *R1*, *R2*, *R3* runner 1, runner 2, runner 3; *B1* box stable 1; *B2* box stable 2; *All R* average for all runners; *All B* average for box stables

The study also involved monitoring PM contamination inside the stables (Tables 9–10). The highest concentration was recorded for PM_10_ fraction and in runners amounted to 177 µg/m^3^ BB and to 368 µg/m^3^ AB; in box stables, dustiness was slightly lower (Tukey’s test, *p* < 0.05). In both types of stables, AB dustiness increased: from 166.3 to 208% depending on the fraction for runners, and for boxing stables from 135.6 to 180.4% (Tukey’s test, *p* < 0.05). The dustiness of the air in the stables in relation to the atmospheric air at the control site was at most several times higher.

**Table 9:** Particulate matter (PM) divided into fractions (µg/m^3^)

Particulate matter fractions	Runners (R1–R3)				Change in relation to the average particulate matter fraction concentrations (%)
	BB		AB		
	Average ± standard deviation	Range	Average ± standard deviation	Range	
PM10	177b***** ± 115	39–636	368a ± 234	64–1110	208.0
PM4	155b ± 73	36–298	276a ± 143	54–592	178.4
PM2.5	143b ± 73	33–314	230a ± 116	49–454	160.4
PM1	135b ± 66	28–258	225a ± 162	39–847	166.3
Box stables (B1–B2)					
PM10	169b ± 85	49–370	305a ± 153	140–740	180.4
PM4	156b ± 76	43–318	254a ± 88	120–412	162.4
PM2.5	148b ± 71	38–282	216a ± 78	110–387	146.2
PM1	134b ± 65	32–244	182b ± 68	66–321	135.6
Control (C)					
PM10	117c ± 53	41–191			
PM4	115c ± 56	43–189			
PM2.5	117c ± 61	42–196			
PM1	107c ± 61	31–198			

Legend: *BB* before bedding, *AB* after bedding; *PM10* PM particles not larger than 10 μm, *PM4* PM particles below 4 μm, *PM2.5* PM particles below 2.5 μm, *PM1* PM particles below 1 μm

^*****^Averages marked with the same letters are not significantly different by Tukey’s test (*p* > 0.05)

**Table 10:** Average particulate matter (PM) concentrations (average calculated from all particulate matter fractions) depending on the season, the horse care system—before (BB) and after bedding (AB) (µg/m^3^)

Stable	Season							
	Spring		Summer		Autumn		Winter	
	BB	AB	BB	AB	BB	AB	BB	AB
R1	146b ******	171b	216a	244a	151c	205b	123c	350a
R2	139b	234a	162b	243a	160c	282a	121c	263b
R3	160b	365a	154b	148b	174c	357a	124c	434a
**All R**	**148b**	**257a**	**177b**	**211a**	**162c**	**281a**	**123c**	**349a**
B1	134b	170b	158b	248a	168c	248a	127c	227b
B2	128b	174b	178b	188b	187c	271a	132c	388a
**All B**	**131b**	**172b**	**168b**	**218a**	**178c**	**259a**	**130c**	**308a**
**C**	120		56		141		140	

Legend: *BB* before bedding; *AB* after bedding; *R1*, *R2*, *R3* runner 1, runner 2, runner 3; *B1* box stable 1; *B2* box stable 2; *All R* average for all runners; *All B* average for box stables, *C* control

^******^Averages marked with the same letters are not significantly different by Tukey’s test (*p* > 0.05)

The calculations of the average PM concentrations in the stables shown in Table 11 were made assuming that the average PM concentration at the control site is constant irrespective of the season and amounts to 100 µg/m^3^. Taking into account this criterion, the lowest PM level for all studied stables BB was recorded in the winter. It was found that the air in the winter in all stables contains less particles than the control site. The lowest PM concentration was observed in the spring AB in almost all stables. The heaviest air contamination with PM was recorded in the summer. The lowest PM increase caused by bedding was observed in the summer—by 19% in runners and by 30% in box stables, on average. Contradictory results were obtained for the measurements taken in the winter. In that case, an increase in PM concentration amounted to 185% and 137%, respectively.

**Table 11:** Relative average particulate matter concentration in the stables (assuming that at the control, it is constant in all seasons and amounts to 100 µg/m^3^) and the change in concentration between BB and AB

Stable	Season											
	Spring			Summer			Autumn			Winter		
	BB (µg/m^3^)	AB (µg/m^3^)	Ch (%)	BB (µg/m^3^)	AB (µg/m^3^)	Ch (%)	BB (µg/m^3^)	AB (µg/m^3^)	Ch (%)	BB (µg/m^3^)	AB (µg/m^3^)	Ch (%)
R1	122	142	17	389	438	13	107	145	36	88	250	183
R2	116	195	68	292	437	50	114	200	76	86	188	118
R3	133	304	129	277	267	− 3	123	253	105	88	310	251
**All R**	**124**	**214**	**73**	**319**	**381**	**19**	**115**	**200**	**74**	**88**	**249**	**185**
B1	112	141	26	284	446	57	119	176	47	91	162	79
B2	107	145	35	320	338	6	133	192	45	95	277	193
**All B**	**109**	**143**	**31**	**302**	**392**	**30**	**126**	**184**	**46**	**93**	**220**	**137**
**C**	**100**											

Legend: *BB* before bedding; *AB* after bedding; *Ch* change of particulate matter concentration between measurements: BB and AB; *R1*, *R2*, *R3* runner 1, runner 2, runner 3; *B1* box stable 1; *B2* box stable 2; *All R* average for all runners; *All B* average for box stables; *C* control

The lowest average temperatures were recorded in the winter, while the highest in the summer (Table 12). The difference between the temperatures measured within runners and box stables did not exceed 1.3 °C. Higher temperatures were observed in runners in the spring, summer, and autumn. Temperature variations inside studied stables were usually minor and reached up to 5 °C only in the spring. The values obtained for relative humidity in the spring and winter were comparable and fell within the range of 43.9–74.7%. The highest relative humidity was recorded for autumn measurements. No significant differences between relative humidity in two types of stables were detected. Relative humidity increased by few percent, as compared with the control site in three seasons—spring, autumn, and winter. As a counterbalance, in the summer, humidity inside the stables was several times higher. However, statistical analysis failed to indicate any significant effect of microclimate parameters (temperature and humidity) on the bacterial aerosol concentration (Pearson correlation, *p* > 0.05).

**Table 12:** Microclimatic parameters depending on the season and the horse care system

Season	Type of research point	Microclimatic parameters			
		Temperature (°C)		Relative humidity (RH) (%)	
		Average ± standard deviation	Range	Average ± standard deviation	Range
Spring	All R	14.8 ± 4.8	8.9–23.0	57.5 ± 6.7	43.9–68.5
	All B	15.3 ± 4.9	10.6–21.0	56.3 ± 6.3	46.3–64.7
	C	15.3 ± 5.3	11.5–19.0	63.5 ± 8.5	57.5–69.5
Summer	All R	25.1 ± 2.0	23.2–29.6	72.8 ± 7.1	57.8–83.7
	All B	26.4 ± 1.7	24.2–29.3	69.9 ± 6.1	57.5–76.4
	C	28.4 ± 2.3	26.8–30.0	58.9 ± 12.9	49.7–68.0
Autumn	All R	11.2 ± 1.1	9.9–12.7	72.7 ± 2.6	68.3–75.7
	All B	12.2 ± 1.3	11.0–13.4	72.0 ± 7.4	61.2–77.3
	C	10.1 ± 1.6	8.9–11.2	83.3 ± 4.2	80.3–86.2
Winter	All R	5.9 ± 1.5	3.1–7.5	57.2 ± 7.8	46.4–74.7
	All B	5.5 ± 1.4	3.1–7.0	57.9 ± 4.0	51.6–62.4
	C	− 3.5 ± 2.1	− 5.0- − 2.0	64.9 ± 21.7	49.5–80.2

Legend: *All R* average for all runners, *All B* average for box stables, *C* control

Positive correlations were found between individual bioaerosol and PM fractions (Table 13). It is common knowledge that bacteria is transmitted through the air not itself but thanks to particles. The results presented here confirm this fact, as we have established a positive correlation between bioaerosol and PM fractions (Pearson correlation, *p* < 0.05). It means that the majority of PM inside studied livestock facilities constituted biological particles, not dust particles (grain dust). An interesting fact is that the study documented a negative correlation between the temperature and all PM fractions.

**Table 13:** Correlations between the examined parameters

	F1	F2	F3	F4	F5	F6	RF	TC	PM10	PM4	PM2.5	PM1	Temp
F2	0.68												
F3	0.77	0.85											
F4	0.77	0.82	0.93										
F5	0.68	0.67	0.80	0.83									
F6	0.57	0.70	0.71	0.70	0.83								
RF	0.75	0.81	0.92	0.93	0.95	0.88							
TC	0.80	0.85	0.94	0.94	0.93	0.87	0.99						
PM10	0.36	0.39	0.32	0.34	0.19	0.23	0.28	0.32					
PM4	0.34	0.31	0.29	0.30	0.18	0.28	0.28	0.30	0.89				
PM2.5	0.24	0.23	0.17	0.19	0.08	0.18	0.16	0.19	0.69	0.88			
PM1	0.17	0.16	0.14	0.14	0.08	0.17	0.14	0.15	0.84	0.87	0.72		
temp	− 0.08	− 0.03	0.04	− 0.01	0.02	0.07	0.03	0.01	− 0.33	− 0.35	− 0.40	− 0.34	
RH	− 0.17	− 0.08	− 0.06	− 0.04	0.04	0.04	0.00	− 0.03	− 0.11	− 0.03	− 0.02	− 0.04	0.22

Legend: Fractions of bacterial aerosol: F1 = 11.0–7.0 µm, F2 = 7.0–4.7 µm, F3 = 4.7–3.3 µm, F4 = 3.3–2.1 µm, F5 = 2.2–1.1 µm, F6 = 1.1–0.65 µm

*TC* total count; *RF* respirable fraction; *PM10* PM particles not larger than 10 μm; *PM4* PM particles below 4 μm; *PM2.5* PM particles below 2.5 μm; *PM1* PM particles below 1 μm; *Temp* temperature of air; *RH* relative humidity

## Discussion

The concentration of airborne microorganisms inside the stables depends on many factors, among others, type of horse management system, feeding system and bedding, the number of horses, feeding schedule, horses’ health, and the surface-area-to-volume ratio (Budzińska et al. 2016). In the case of stables analyzed in this study, the surface-area-to-volume ratio ranged between 71.8 and 96.7 for runners, while for box stables, it fell within the range of 113.0–125.1. On the other hand, the “volume per 1 animal” index ranged from 65.92 to 92.7 m^3^/1 animal (horse) in runners and 81.07 to 81.4 m^3^/1 animal (horse) in box stables. Kośla [2001] gives the cubature index (“volume per 1 animal”) in the range of 24–45 m^3^/1 horse, while Kaletowski [1997] 30–35 m^3^/1 horse; therefore, the studied stables are about three times better. The value of this index given by Bombik et al. [2009] is 39.1 m^3^/1 horse in stationary stables and 37.3 m^3^/1 horse in box stables.

In stables, similarly to other livestock facilities, the concentration of bacteria was few to several dozen times higher as compared with residential buildings. It depends on multiple factors and the most important ones include animal density, type of feed, and the presence of feces. Despite the fact that admissible concentrations of airborne microbial contamination seem to be high, they were exceeded in the case of 1/5 of TC measurements for the bacterial aerosol and for more than 1/4 of measurements for the bioaerosol RF. In our study, the recommended bioaerosol concentration was exceeded most frequently in the spring, and thus, the highest concentrations were recorded in that season too (Table 3). These levels were slightly lower in the summer, which means that warm seasons are distinguished by worse bacteriological conditions. Witkowska et al. (2012) detected the highest bioaerosol concentrations in the summer. The occurrence of the elevated bioaerosol concentrations in warm periods is understandable, as an increased temperature and suitable humidity stimulate bacterial growth and their migration into the air (Perrin 2021). Seasonal variations in bacteria concentration were confirmed by Budzińska et al. (2016). Bacterial concentrations from the spring to autumn fell within the range of 104,000–590,000 CFU/m^3^, while the concentrations recorded in the winter were one order of magnitude lower. Samadi et al. (2009) detected very low bioaerosol concentrations in the stables. Our results were several dozen times higher as compared with the measurements obtained by these authors. On the other hand, bacterial concentrations measured by Witkowska et al. (2012) covered a lot wider range, with our results fell within that limits. Even higher concentrations were detected by Sowińska et al. (2015) who conducted research in box stables during autumn and winter: 447,000–1,175,000 CFU/m^3^.

As mentioned above, PM and bioaerosol concentrations depend to a large extent on the type of bedding used in the stable. It translates into the obtained results—bioaerosol concentrations in runners, where the bedding was replaced less frequently, were significantly higher than in box stables. The differences reached 250%. Fleming et al. (2008) obtained the lowest bioaerosol concentrations when straw pellet was used as bedding. Irrespective of the material applied as bedding in the stable, the authors revealed a significant increase of the airborne particle concentration during cleaning the stable, especially when the straw was used. As reported by Clauβen and Hessel (2017), the lowest concentrations for both bioaerosol and PM were detected when the boxes were thoroughly cleaned from the straw and excrement on a daily basis. Higher concentrations were obtained when additional straw layers were added and feces were not removed. These observations confirm the results obtained in the course of our study.

The seasons of the year may affect the number of microorganisms and their spread in the air. Witkowska et al. (2012) suggests that high bacterial contamination of the air in the winter inside the stables results from poor ventilation (closed doors and windows), while higher contamination levels in the summer are probably associated with increased temperatures and humidity that encourage microbial growth.

There is no literature data available regarding bioaerosol fraction and the proportion of respirable fraction to compare with the results obtained in this study. We can only make a comparison with the results delivered in the studies conducted in zoological gardens concerning similar animals, e.g., giraffes or camels. The air in the facilities dedicated for giraffes in the zoological garden in Cracow was characterized by a different grain distribution—higher parentage shares were obtained for larger particle fractions and it amounted to 59% for the respirable fraction, while in the stables after cleaning (AB—these conditions were applied during carrying out research in zoological gardens), it reached the value of 68% (Grzyb and Lenart-Boron 2019). Contradictory results were obtained in the facilities in other zoological garden—in Chorzów. In that case, RF share was even higher and amounted to 74% for giraffes and as much as 86% for camels (Grzyb and Pawlak 2021).

As far as the problem of bacterial intoxication inside livestock facilities is concerned, it has not been studied and explained in detail yet. Available literature provides data regarding a vet clinic (Bulski et al. 2019) or facilities for other livestock animals, but not horses. Matković et al. (2007) studied intoxication occurrence in the stable and reported that bioaerosol concentrations indoors were 73 to 102 times higher depending on the time of the day.

Polish legislation provides for the admissible concentration of PM_10_ and PM_2.5_ in the atmospheric air. Direct comparisons can be made only with regard to a limit value for PM_10_, as the value indicated for fraction PM_2.5_ regard the whole calendar year. The maximum admissible PM_10_ concentration amounts to 50 µg/m^3^, which means that permissible PM_10_ concentration in the studied stables was exceeded at all times, especially after cleaning. On the other hand, Fiedorowicz (2007) claims, based on animal hygiene handbooks, that the maximum PM concentration inside the stable cannot exceed 3 mg/m^3^. In that case, permissible particulate contamination in the studied facilities was not exceeded.

The evaluation of the PM content inside the stables revealed that the highest concentrations AB occur in the summer and autumn, while BB in the winter (Table 10). However, when the PM concentration is standardized to the control site (Table 11), the concentration changes. The highest concentration of PM was observed in the summer and it was several dozen percent higher than in other seasons of the year. High PM concentrations in a warm season were not compensated by better ventilation supported by opening doors and windows in the stables. Poor ventilation in the winter was reflected in the indicator of the changes in PM concentration BB in relation to AB. Similar patterns were noticed by Elfman et al. (2009).

Riihimäki et al. (2008) reported approximately 3–4 higher PM concentrations in box stables for racehorses in Sweden, as compared with the results obtained in our study. Millerick-May et al. (2013) reported that in high stables in the breathing zone both for groomers and horses, the concentration of PM is lower—both for PM_10_ and PM_2.5_ fractions. It results from more efficient ventilation and greater air volume inside the stable.

Clauβen and Hessel (2017) suggest that using wet cleaning systems with opening doors and windows has a significant impact on the particulate level in box stables.

The results delivered by Wålinder et al. (2011), who measured two PM fractions: total PM (PM_10_) and respirable PM (PM_4_), are consistent with the data obtained in our study for the runner BB. The measurements made by Wålinder et al. (2011) fell within the following ranges—for PM_10_: 100–790 µg/m^3^ (mean 210 µg/m^3^), and for PM_4_: 40–410 µg/m^3^ (mean 100 µg/m^3^).

The studies undertaken by Clements and Pirie (2007) on the correlation between PM contamination and the type of bedding and feed produced very interesting results. The lowest concentrations of the respirable fraction (PM_4_) were recorded when wood shavings were used as a bedding material and animals were fed with silage (the mean PM concentration amounted to as little as 26 µg/m^3^). When the straw was used as bedding and the horses were provided with hay as fodder, PM concentration amounted to 87 µg/m^3^, on average. Siegers et al. (2018) applied the same bedding/fodder pattern in runners and reported that PM_10_ concentration amounted to 140 µg/m^3^ for the first setup and 1100 µg/m^3^ for the second one.

Our results are in conflict with the ones delivered by Wolny-Koładka (2018). The author conducted research in 2 box stables and reported a significant increase (even sixfold) in PM concentration in the winter, as compared to other seasons.

Microclimatic conditions, temperature and relative air humidity, exert considerable influence on the horses’ health and well-being. Exceeding optimum values for these parameters may result in deteriorating both physical and mental condition of the horses (Budzińska et al. 2016). Adult horses in comparison with foals display higher tolerance to low temperatures (Kołacz and Dobrzański 2019; Kośla and Porowska 2013). According to Kośla (2011), the minimum temperature inside the stable for adult horses should not fell below 4–6 °C. In our study, the minimum temperature was slightly lower and amounted to 3.1 °C, while the maximum temperature recorded in the summer reached almost 30 °C. The temperatures measured by Kośla and Porowska (2013) were comparable; however, the minimum temperature was moderately lower (reached 2.5 °C), while the maximum was almost the same. Bombik et al. (2011), who conducted research in box stables in Mazury in the spring, recorded temperatures covering the following range: 8.5–14.4 °C; a temperature range in our study was significantly broader (8.9–21.0 °C).

As far as our study is concerned, the average temperature was higher in box stables—in the period from the spring to autumn (0.5–1.3 °C), while in the winter, higher temperatures were detected in runners (by 0.4 °C). Slightly different results were obtained in the study carried out by Kwiatkowska-Stenzel (2011), who recorded lower temperatures in runners than in box stables throughout the entire year. Bombik et al. (2009) reported a significantly higher temperature difference in the winter between different types of stables: the temperature in a stationary stable was lower by 4 °C as compared to a box stable.

Relative humidity recorded in our study in the stables fell within the following range: 43.9–83.7%. The measurements of relative humidity made by Bombik et al. (2009) covered similar range (50.7–89.1%), with few percent higher upper limit. Pursuant to the Regulation of the Minister of Agriculture and Rural Development of 2017, relative humidity shall not exceed 80%. When it comes to our study, this threshold was only slightly exceeded. Higher maximum relative humidity was recorded by Sowińska et al. (2015)—it reached as much as 92.26%. Relative humidity inside the stables in relation to the control site in the summer was higher by several percent. However, Budzińska et al. (2016) demonstrated a similar correlation in the winter. The author suggests that it depends on the shape of the stables and the quality of the building insulation. To sum up, most microclimate measurements meet animal hygiene standards.

## Conclusions

Based on the results obtained in the course of a 2-year study, we can confirm that both airborne bacterial and particulate contamination are higher in runners than in box stables. A statistically significant correlation between all studied particulate matter and bioaerosol fractions was found. Recommended bioaerosol concentrations were exceeded three times more frequently in runners. High bioaerosol concentrations were recorded mostly in the spring. The activities performed by the staff in the stables, especially cleaning and feeding horses, have a significant impact on the level of the studied particles in the air. Bioaerosol concentrations correlated with microclimatic conditions (temperature and humidity) and were subject to seasonal fluctuations. It is worth highlighting that temperature and humidity in most cases fell within the limits that ensure good animal welfare. We must be aware that exposure of animals, groomers, and other people having contact with horses to increased PM and bacterial contamination may affect their health. Thus, more frequent bedding replacement, especially in the runners, and the application of wet cleaning systems should be taken into consideration. It is worth considering changing the straw storage conditions under roofed sheds, or the use of alternative bedding materials (e.g., wood pellets).

## Data Availability

The datasets used and/or analyzed during the current study are available from the corresponding author on reasonable request.
